# Comparative Quality Indicators for Hospital Choice: Do General Practitioners Care?

**DOI:** 10.1371/journal.pone.0147296

**Published:** 2016-02-03

**Authors:** Marie Ferrua, Claude Sicotte, Benoît Lalloué, Etienne Minvielle

**Affiliations:** 1 EHESP-MOS (École des hautes études en santé publique – Management des organisations de santé), Institut Gustave Roussy, 114, rue Édouard-Vaillant 94805, Villejuif, France; 2 Département d'administration de la santé, Université de Montréal, C.P. 6128, Succ. Centre-ville, Montréal, Québec, H3C 3J7, Canada; Azienda Ospedaliero-Universitaria Careggi, ITALY

## Abstract

**Context:**

The strategy of publicly reporting quality indicators is being widely promoted through public policies as a way to make health care delivery more efficient.

**Objective:**

To assess general practitioners’ (GPs) use of the comparative hospital quality indicators made available by public services and the media, as well as GPs’ perceptions of their qualities and usefulness.

**Method:**

A telephone survey of a random sample representing all self-employed GPs in private practice in France.

**Results:**

A large majority (84.1%–88.5%) of respondents (n = 503; response rate of 56%) reported that they never used public comparative indicators, available in the mass media or on government and non-government Internet sites, to influence their patients’ hospital choices. The vast majority of GPs rely mostly on traditional sources of information when choosing a hospital. At the same time, this study highlights favourable opinions shared by a large proportion of GPs regarding several aspects of hospital quality indicators, such as their good qualities and usefulness for other purposes. In sum, the results show that GPs make very limited use of hospital quality indicators based on a consumer choice paradigm but, at the same time, see them as useful in ways corresponding more to the usual professional paradigms, including as a means to improve quality of care.

## Context

In response to the array of challenges faced by healthcare systems today, the strategy of publicly reporting quality indicators is being widely promoted through public policies as a way to make health care delivery more efficient. This approach has resulted in the development and public reporting of many quality indicators that emphasize a more quantitative approach to management, one based on extensive use of the kind of indicators found in quality management and performance measurement models. This trend has become the trademark of various public policies known as New Public Management (NPM). [1–2]

NPM has become very popular in countries in which the government owns and operates healthcare facilities and services. NPM is particularly notable for efforts to achieve greater governance transparency based on public reporting, thereby encouraging behaviours through free market effects. Public quality report cards–an idea borrowed from more private healthcare systems such as that in the U.S.–represent an attempt to trigger new mechanisms in the public domain. Greater efficiency in the delivery of health care is sought by treating patients as consumers, having them make more choices about their health providers to generate more competition between healthcare facilities. [3–7]

With this vision in mind, patients are provided with comparative information, such as indicators measuring the quality of various care providers or different healthcare facilities. Patients, who are seen as consumers, are expected to actively participate in the selection of their health providers in order to benefit from superior treatment opportunities. [3,5–10] These behaviours should, in theory, encourage health providers to deliver better quality care in order to attract such consumer patients. The idea is that healthy competition, based on reputation and quality, will emerge. However, the potential benefits of such a public health policy are based on demanding requirements, such as the willingness and ability of patients to use this publicly reported information in their decision making. Regarding this, several studies have revealed major barriers to the use of comparative information and have highlighted patients’ limited expertise. [4,6,10–13]

However, such a policy of public reporting could still be effective if physicians are sufficiently interested in comparative information to use it as a basis for influencing their patients’ choices. Healthcare decisions remain a complex subject, and patients faced with limited information, will often delegate the choices to their physician. [4,10,14] Public reporting efforts have also been based on such reasoning, which is why they are directed at healthcare professionals in some countries; a trend less evident in the US where report cards, for the most part, are still clearly intended for consumers. However, here again the comparative information will only be useful if physicians consider it valid and usable. The available studies showed that specialists were skeptical about public reporting, and considered the reported indicators to be of limited use. [3,4,8,15] Similarly, the results of four studies analyzing the attitudes of GPs point in the same direction. In one study, informal sources of information such as geographical proximity or comments from patients previously referred were more important than comparative quality indicators. [14] Another large-scale study with a random sample taken from the entire population of GPs in Scotland showed that comparative clinical performance data were far from being the main source used to assess the hospital trusts. [16] Besides, a very weak effect of comparative indicators in the referral patterns of GPs was found in a study comparing three different diagnoses. [15] Finally, a study based on a random sample of 300 GPs concluded that Germany’s mandatory hospital quality reports played only a minor role in physicians’ counseling of patients who needed hospital care because too few physicians knew about and used the reports. [17]

In France, publicly reporting comparative indicators made its way into French national policy in 2008. Even if the adoption of NPM policies in the French healthcare system has not been as extensive as in Anglo-Saxon countries like the U.K. or Australia, the number of policies inspired by the NPM movement has been steadily increasing. [18] For instance, France introduced a preferred doctor scheme in 2006 to give a larger role to GPs as gatekeepers, providing patients with access to medical specialists and hospital care. [19–20] According to this scheme, the GPs did not receive a concrete monetary incentive for referring a patient, but the patient paid more if he or she did not consult a GP to be referred to specialized care.

Given these trends and the mixed evidence with respect to GPs, the objective of this study was to measure GPs’ use and perceptions of the comparative information provided to them by public services and the media. To this end, we conducted a broad national survey of French GPs.

## Methods

### Study population

In the spring of 2014, a telephone survey of a random sample representing all the general practitioners (GPs) of France was conducted. This sample was extracted from a national database that identifies all French self-employed GPs in private practice. As of January 1, 2013, this target population consisted of 54,579 GPs. An approximately 1% random sample drawn from this database was used to identify a sample of 1000—plus an extra 230—GPs based on the prediction of a 50% survey response rate. We sought a sufficiently large number to produce valid descriptive statistics on GPs’ attitudes (with a margin of error of approximately 5%) while limiting the number of respondents for logistical reasons. The physicians were initially contacted by email and then telephone by a public research institution specialized in this kind of survey (*Observatoire Régional de la Santé*, Provence-Alpes-Côte d’Azur). Each study participant provided verbal informed consent to participate in the survey. The verbal consent was registered by the research institution conducting the survey. This consent procedure was approved by an ethics approval commission. The physicians who participated in the study received monetary compensation in an amount equal that paid for a medical consultation.

### Survey questionnaire

A literature review was conducted to build a comprehensive questionnaire, including all the themes typically addressed in such studies. An association of GPs in private practice was consulted in order to validate the questionnaire’s dimensions and terminology. The questionnaire was also pre-tested with ten GPs. The closed questionnaire (cf. S1 File) was organized around three themes:

1 The frequency of use of various sources of information that can support the selection of a hospital.

Two types of public sources were covered: formal sources such as the French health department website (publicly reported hospital quality indicators developed by health services researchers) [21]; and the mass media (L’Express, Le Point) and websites popular with the general public (e.g. Doctissimo). Also, informal sources were assessed such as professional relationships, personal experience, word of mouth, geographical proximity and patient preferences (Table 1). The website of France’s department of health (called *PLATINES* at the time, now called *Scope santé*) had, since 2004, offered benchmarking information to the general public on the activities and quality of French health facilities. The site presents results for care quality and safety indicators (e.g. the control of nosocomial infections, patient recordkeeping, pain assessment, etc.) based on data that each health facility is required to collect. These results are expressed as a score, a performance class (A, B and C) determined by the score’s ranking, and its confidence interval given the performance thresholds defined by health authorities. Also, the French media has been comparing and rating he nation’s health facilities since 1998. These ratings are established using a wide range of methodologies and built from aggregate scores primary drawn from health-administrative data (activity, attractiveness, technological sophistication, length of stay, complexity, etc.), self-reported data (equipment, human resources, etc.) and quality indicator results.

**Table 1:** General practitioners’ frequency of use of different information sources for hospital choice.

	Never	Rarely	Sometimes	Often	Always	Do not know
To guide your patient to a hospital, at which frequency do you use:	%	%	%	%	%	%
	(95% Confidence interval)	(95% Confidence interval)	(95% Confidence interval)	(95% Confidence interval)	(95% Confidence interval)	(95% Confidence interval)
Public authorities’ quality indicators?	87.9	6.2	3.4	2.2	0.0	0.4
	(84.7–90.6)	(4.2–8.6)	(2.0–5.4)	(0.0–4.8)	(0.0–0.7)	(0.0–1.4)
Media (hospital league tables)?	84.1	9.9	4.4	1.4	0.0	0.2
	(80.6–87.2)	(7.5–12.9)	(2.8–6.5)	(0.6–2.8)	(0.0–0.7)	(0.0–1.1)
Nongovernmental websites?	88.5	6.4	4.0	1.0	0.0	0.2
	(85.3–91.1)	(4.4–8.9)	(2.4–6.1)	(0.3–2.3)	(0.0–0.7)	(0.0–1.1)
Informal personal networks?	4.2	1.6	6.6	42.7	44.7	0.2
	(2.6–6.3)	(0.7–3.1)	(4.6–9.1)	(38.4–47.2)	(40.3–49.2)	(0.0–1.1)
Previous personal experience?	1.8	1.0	3.8	48.5	43.9	1.0
	(0.8–3.4)	(0.3–2.3)	(2.3–5.8)	(44.1–53.0)	(39.5–48.4)	(0.3–2.3)
Word of mouth?	2.2	2.8	9.9	49.9	34.6	0.6
	(1.1–3.9)	(1.5–4.6)	(7.5–12.9)	(45.4–54.4)	(30.4–38.9)	(0.1–1.7)
Geographical proximity?	1.0	3.2	10.1	50.9	34.8	0.0
	(0.3–2.3)	(1.8–5.1)	(7.6–13.1)	(46.4–55.3)	(30.6–39.1)	(0.0–0.7)
Patient preference?	2.0	4.2	24.1	49.9	19.9	0.0
	(1.0–3.6)	(2.6–6.3)	(20.4–28.0)	(45.4–54.4)	(16.5–23.6)	(0.0–0.7)

2 Perceptions of the quality (validity, relevance and interpretability) and the usefulness (quality of care and transparency of the public authorities) of publicly reported indicators (Table 2).
3 The value of five common publicly reported indicators on hospitals (Table 3). The French healthcare authorities were interested in these quality indicators. Indeed, studies had been sponsored by the Ministry of Health and the *Haute Autorité de Santé* (HAS–National Authority for Health) to confirm their validity. [22]

**Table 2:** General practitioners’ opinions on the quality and usefulness of hospital quality indicators.

What is your opinion about the following statements?	Strongly agree	Agree	Indifferent	Disagree	Strongly disagree	Do not know
	%	%	%	%	%	%
	(95% Confidence interval)	(95% Confidence interval)	(95% Confidence interval)	(95% Confidence interval)	(95% Confidence interval)	(95% Confidence interval)
**Perceptions of quality**						
Valid for rating quality of care	3.4	33.6	19.9	20.5	7.6	15.1
	(2.0–5.4)	(29.5–37.9)	(16.5–23.6)	(17.0–24.3)	(5.4–10.2)	(12.1–18.5)
Useful for choosing a hospital	4.8	37.8	15.3	25.0	6.4	10.7
	(3.1–7.0)	(33.5–42.2)	(12.3–18.8)	(21.3–29.1)	(4.4–8.9)	(8.2–13.8)
Easy to understand	4.6	32.2	15.3	20.1	3.4	24.5
	(2.9–6.8)	(28.1–36.5)	(12.3–18.8)	(16.7–23.9)	(2.0–5.4)	(20.8–28.5)
**Usefulness**						
Improves quality of care	8.0	49.7	11.3	9.3	2.2	19.5
	(5.7–10.7)	(45.2–54.2)	(8.7–14.4)	(6.9–12.2)	(1.1–3.9)	(16.1–23.2)
Improves the transparency of public policies	2.7	38.7	12.7	19.2	5.9	20.8
	(1.4–4.5)	(34.3–43.1)	(9.9–16.0)	(15.8–23.0)	(4.0–8.4)	(17.3–24.7)

**Table 3:** General practitioners’ opinions on the utility of common hospital quality indicators.

For you, is it important to have data about:	Strongly agree	Agree	Indifferent	Disagree	Strongly disagree	Do not know
	%	%	%	%	%	%
	(95% Confidence interval))	(95% Confidence interval)	(95% Confidence interval)	(95% Confidence interval)	(95% Confidence interval)	(95% Confidence interval)
Pressure ulcer rates?	25.4	47.4	7.2	13.5	2.0	4.5
	(21.6–29.5)	(42.9–52.0)	(5.0–9.8)	(10.6–16.8)	(1.0–3.7)	(2.8–6.7)
Practitioner quality?	11.9	35.2	12.9	20.7	7.4	12.0
	(9.1–15.1)	(30.9–39.6)	(10.0–16.2)	(17.2–24.5)	(5.2–10.0)	(9.3–15.3)
Quality of coordination between hospital and primary care?	26.2	55.3	6.8	7.2	1.4	3.2
	(22.4–30.1)	(50.8–59.7)	(4.7–9.3)	(5.1–9.8)	(0.6–2.8)	(1.8–5.1)
Patients’ satisfaction?	21.3	54.1	9.1	10.5	2.0	3.0
	(17.8–25.1)	(49.6–58.5)	(6.8–12.0)	(8.0–13.6)	(1.0–3.6)	(1.7–4.9)
Mortality rates?	11.3	36.2	14.3	25.2	7.2	5.8
	(8.7–14.4)	(32.0–40.6)	(11.4–17.7)	(21.5–29.3)	(5.1–9.8)	(3.9–8.2)

All measures used a 5-point Likert scale.

Ethics approval for this research was obtained from the CNIL (*Commission nationale de l'informatique et des libertés*).

## Results

Among the 1,230 targeted GPs, 900 (73.2%) were personally contacted. The rest, who could not be contacted after 15 telephone calls on different days and at different times, were classified as unreachable. Among these 900 GPs, 503 agreed to complete the questionnaire (a response rate of 56%). No significant difference was observed between these 503 respondents and the entire study population (n = 54,579) (Fisher’s exact test for sex, age, region of graduation and type of practice: solo, group or in partnership). Most were men (67.4%), aged between 50 and 64 years (63%), and in solo practice (52.9%). However, the non-respondents (n = 397) included a significantly higher proportion of men (74.1%) (p = 0.03) and slightly older GPs (p = 0.008).

Table 1 describes the frequency of use of various public sources that could justify the choice of a given hospital. The use of public quality indicators was low. A large majority of GPs reported that they never used these indicators (84.1%–88.5%). In contrast, they often used traditional sources such as personal experience (often/always, 92.4%), informal networks (87.4%), geographical proximity (85.7%), word of mouth (84.5%) and patient preferences (69.8%).

The GPs had mixed opinions about the qualities associated with publicly reported comparative information (Table 2). The largest group of GPs (37% to 43%) expressed a favourable opinion (strongly agree/agree). However 23%–31% expressed negative opinions while 15%–20% remained ambivalent (Likert scale mid-point). The remaining GPs (11% to 25%) were unaware of comparative indicators. In addition, Table 2 shows the usefulness of publicly reporting hospital quality indicators to meet the two major objectives of public policy. The largest proportion (57.7%) of favourable opinions (strongly agree/agree) was observed for improving quality of care, while improved transparency was reported by 41.4% of the GPs. But, here again, about a third of the respondents (30%–32%) did not have a clear opinion. In sum, high proportions of GPs reported favourable opinions about public comparative information–both in terms of its quality and its usefulness (Table 2)–despite the fact that many reported rarely or never using this information for hospital choice.

Table 3 presents GPs’ views on the value of five common hospital quality indicators. They considered them very important (strongly agree/agree) in proportions varying from 47% (practitioner quality) to 82% (coordination between primary care and hospital care). Here again, the results stood in stark contrast with the use of quality indicators for hospital choice. Despite the fact that the GPs said that they made limited use of public information for hospital choice (Table 1), they nevertheless considered several indicators highly useful (Table 3). So it seems that GPs do not reject off hand the usefulness of comparative information. Rather, it would appear that they see it as useful in ways other than those promoted by the proponents of NPM, who seek to create consumption behaviours inspired by free market principles.

## Discussion

This study sought to assess the opinions of French GPs in private practice about the use and usefulness of publicly reported hospital quality indicators. The strategy of publicly reporting such indicators is widely promoted by public policies that seek to make health care delivery more efficient, a vision often associated with the principles advanced by NPM.

This study has produced two main results. First, GPs made very limited use of comparative indicators when advising their patients. The vast majority of GPs relied mostly on informal sources of information. These results are consistent with similar studies conducted with both GPs [14–17] and specialists. [3,4,7,8] Also, only two other studies have been conducted on a national scale, and they confirm the results of this study in terms of having found very limited use of hospital comparative indicators. [16,17]

The second main result of this study is that, paradoxically, despite the physicians’ low use of comparative information, their perceptions of such indicators were significantly positive. The study found favourable opinions among a large proportion of GPs of several aspects of hospital indicators, such as the quality of the information and its usefulness in terms of quality of care and the state’s transparency with the public with regards to this information. There is a discrepancy between this second result and our first main result. One explanation may be that many GPs had limited knowledge of public quality indicators and had an idealized view of such indicators. Another explanation may be that, although the GPs were quite familiar with the public indicators on which they had favourable opinions, they did not use them because they did not like how they were defined or distrusted the sources of data they used. A third explanation is that there was a social desirability bias operating, but this interpretation is implausible since a similar bias would have been observed for the other main result, that of very limited use of public quality indicators.

Overall, the results of this survey suggest that the premises underlying public policies based on free market paradigms do not lead to a magic-bullet solution. Even if a majority of the studies of public report cards showed a positive impact [7], the evidence remains mixed, and the results of this study should also be used with caution. This study suggests that, apart from patients–conceived as consumers–, it is a challenging task to develop and communicate public indicators in an actionable manner for GPs, who represent another important target user group for such public report cards.

Besides, we should not assume that quality indicators are not used for other purposes. In this respect, it is interesting to note the results of a recent systematic review, which concluded that comparative information was effective at stimulating internal continuous improvement efforts within hospitals rather than influencing hospital choice. Here again, the evidence is mixed but it expands the potential utility of such public policies while, once again, it suggests that execution can be a challenge.

Thus, given the challenges inherent in implementing NPM policies to transform healthcare systems, it may prove useful to draw on institutional theories, focusing the analysis on stability rather than change. [1] To interpret the findings of this study in terms of an institutional theory, [23] one needs to view NPM reforms as a challenger paradigm (such as, in this instance, the market paradigm) that is confronted with a pre-existing, dominant paradigm (such as the professional bureaucracy model). [24] In this sense, the main operators–the physicians–remain focused on the organization of clinical care, so they perceive the usefulness of hospital indicators primarily in terms of their ability to improve quality of care. The physicians therefore see their usefulness as sources of comparative information guiding patients’ choices as much less important. This may explain the physicians’ limited interest in using quality indicators for such a purpose. Other researchers have cited this phenomenon to explain why policies inspired by NPM have failed to be adopted by healthcare professionals. [1] Therefore, an alternative path may be to study comparative information based on models inspired by the management of organizations. The action mechanisms then pertain to the internal dynamics of organizations rather than to external market dynamics. For example, benchmarking uses comparative indicators to identify underperforming sectors where improvements are needed. This perspective may lead to more effective use of hospital indicators by encouraging their adoption by physicians, who may find them useful in ways that are better aligned with their clinical professional paradigm.

### Limitations

A study such as this, conducted on an entire population of GPs, does raise some concerns that should be addressed. First, the sample size could have been larger, but it is nevertheless comparable to other, similar studies [15,17], and it is sufficiently large to reflect the opinions of the population studied. The generazibility of the results is thus good for countries in which the government owns and operates healthcare facilities all the more so that the results are congruent with the findings observed in the two other studies that have been conducted on a national scale. [15,17] Second, the significant difference observed between the non-respondents and the entire study population did not introduce a bias because the study analyzes the opinions of the respondents.

## Conclusion

The results of this study show that the basic premises underlying public policies based on consumption behaviours within a free-market paradigm are challenging with respect to one important group of decision-makers: GPs. Given the considerable efforts made to reform healthcare systems, the results provide food for thought on how to achieve more consistent use of hospital quality indicators. The challenge is to develop public report cards that can have an impact on patient referrals while being closer to the professional paradigm, thereby representing a more promising way to leverage transformations of and improvements to health care. It is even more important to conduct such a review at this time, when several public policy initiatives seek to give GPs a much larger role as gatekeepers in order to better coordinate access to specialized care. If such initiatives are to succeed, it is essential to better understand GPs’ attitudes in this area, and under what circumstances they would consider hospital indicators useful.

## Supporting Information

S1 File Survey Questionnaire. (DOCX)
