# Supplementary material for: Comparative Quality Indicators for Hospital Choice: Do General Practitioners Care?
Source: PLoS One. 2016 Feb 3;11(2):e0147296. doi: 10.1371/journal.pone.0147296 (PMC4740419; doi:10.1371/journal.pone.0147296)
Supplement: S1 File — (DOCX) [file pone.0147296.s001.docx]

**APPENDIX: Survey Questionnaire**

| **When choosing a health care organisation for patient referral, how often do you use the following information sources** | | | | | | Never | Rarely | | Sometimes | Often | Always |  |
| --- | --- | --- | --- | --- | --- | --- | --- | --- | --- | --- | --- | --- |
| 1. Health authorities (Ministry of Health, HAS) | | | | | |  |  | |  |  |  |  |
| 2. Magazines (e.g. Express, Le Point, Le Figaro, Le Nouvel  Observateur) | | | | | |  |  | |  |  |  |  |
| 3. Websites and forums (e.g. Doctissimo, Hospiguide, mon-hopital.fr) | | | | | |  |  | |  |  |  |  |
| **When choosing a health care organisation for patient referral, how often do you use the following criteria** | | | | | | Never | Rarely | | Sometimes | Often | Always |  |
| 4. Informal networking with hospital staff | | | | | |  |  | |  |  |  |  |
| 5. Past experience of the hospital | | | | | |  |  | |  |  |  |  |
| 6. Word of mouth (other GPs) | | | | | |  |  | |  |  |  |  |
| 7. Awareness of official referral route (e.g. rare diseases) | | | | | |  |  | |  |  |  |  |
| 8. Distance | | | | | |  |  | |  |  |  |  |
| 9. Preferences of patients or of their families | | | | | |  |  | |  |  |  |  |
| **What is your view on the following statements on quality indicators (QIs)?** | Strongly agree | | Agree | | Indifferent | | | Disagree | | Strongly disagree | No opinion | |
| 10. QI results reflect quality of care in a  health care organisation |  | |  | |  | | |  | |  |  | |
| 11. QI results are useful when choosing a  health care organisation |  | |  | |  | | |  | |  |  | |
| 12. QIs are easy to understand |  | |  | |  | | |  | |  |  | |
| **What is your view on the following statements on the public disclosure of quality indicators (QIs)?** | | Strongly agree | | Agree | | Indifferent | | | Disagree | Strongly disagree | No opinion |  |
| 13. QIs encourage improvement in the  organisation of quality of care in health  care organisations | |  | |  | |  | | |  |  |  |  |
| 14. QIs enhance healthcare transparency | |  | |  | |  | | |  |  |  |  |

| **In your opinion, availability of data on the following criteria is important to gauge the quality of a health care organisation:** | Strongly agree | Agree | Indifferent | Disagree | Strongly disagree |
| --- | --- | --- | --- | --- | --- |
| 15. Pressure ulcer rate |  |  |  |  |  |
| 16. Practitioner excellence |  |  |  |  |  |
| 17. Quality of organisation (e.g . time to send  discharge letter) |  |  |  |  |  |
| 18. Patient satisfaction and experiences |  |  |  |  |  |
| 19. Mortality rate |  |  |  |  |  |

The last few questions concern your practice

| **20. How long have you been a GP?** |
| --- |
| Number of years : _________ |
| **21. Have you a specialty other than general medicine?** |
|  Yes Please specify : ____________________________ |
|  No |
| **22. Are you a member of a peer group or best practice sharing group** |
|  Yes |
|  No |
| **23. How long have you been practising in your area ?** |
| Number of years : _________ |
| **24. How many visits (office and home) do you have/make on average per week ?** |
|  Fewer than 50 |
|  Between 50 and 100 |
|  Between 100 and 150 |
|  More than 150 |
